# An extremely rare case: Transorbital penetrating intracranial injury by wooden foreign body. Case report

**DOI:** 10.1016/j.amsu.2021.102937

**Published:** 2021-10-14

**Authors:** Wahyudi, Ahmad Zaky, Andi Asadul Islam, Prihantono, Rohadi Muhammad Rosyidi

**Affiliations:** a Faculty of Medicine, Muhammadiyah Makassar University, Departement of Neurosurgery, Medical Faculty of Hasanuddin University, Makassar, Indonesia; b Faculty of Medicine, Muhammadiyah Makassar University, Makassar, Indonesia; c Departement of Neurosurgery, Medical Faculty of Hasanuddin University, Makassar, Indonesia; d Departement of Surgery, Medical Faculty of Hasanuddin University, Makassar, Indonesia; e Department of Neurosurgery Medical Faculty of Mataram University, West Nusa Tenggara General Hospital, Mataram, Indonesia

**Keywords:** Wooden foreign body, Penetrating injury, Orbitocranial injury, Multidisciplinary approach, Case report

## Abstract

**Introduction and importance:**

Transorbital Penetrating Intracranial Injury (TOPI) is a rare case, but those caused by Wooden Foreign Body are even challenging that may pose unusual diagnostic and surgical challenges.

**Case presentation:**

we presented a TOPI following a wood penetrated to the left temporal fossa region via orbital roof due to struck the tree branches while got a motor vehicle accident. The patient was fully conscious with decreased visual acuity in the left eye and left ophthalmoplegia. Non-contrast CT scan showed the linear-shaped foreign body, air mimicking that penetrated medial orbit plane to the left temporal fossa.

**Clinical discussion:**

The surgery was performed with a temporobasal approach and revealed good results with only mild ophthalmologic complications without long-term fatal complications (1-year followed-up).

**Conclusion:**

early removal of wooden foreign body that penetrates to the intracranial via transorbital is mandatory and should be involved multidisciplinary approach to get the optimal result and avoid the fatal complication both neurologically or ophthalmologically.

## Highlights

• Transorbital Penetrating Intracranial Injury (TOPI) is rare case.
• We presented a TOPI following a wood penetrated to the left temporal fossa region via orbital roof due to strucked the tree branches while got a motorvehicle accident.
• Early removal of wooden foreign body that penetrate to the intracranial via transorbital is mandatory and should be involved multidisciplinary approach in order to get optimal result and avoid the fatal complication both neurologically or ophtalmologically.

## 1 Introduction

TOPI is one of the rare cases of traumatic brain injury. It is just found only 0,4% of all traumatic injuries with a major cause is the missile such bullet in the war trauma setting. Otherwise, nonmissile foreign bodies such as iron rods, wood, nail, and needle are even scarcer [[1], [2]].

Penetrating Brain Injury are fatal due to damage to important structures such as vascular, brainstem, and also infection can occur. Although infection always occurs in delayed treatment, in the case of TOPI that is caused by wood material, the risk of infection is likely increased. However, early treatment with surgical and nonsurgical approaches can reduce chances of infection and hemorrhage, therefore morbidity and mortality significantly [1,3,4]. Here we present an extremely rare case of transorbital penetrating intracranial injury by a wooden foreign body with a quite satisfactory result without fatal long-term complication.

## 2 Case report

**Fig. 1:**
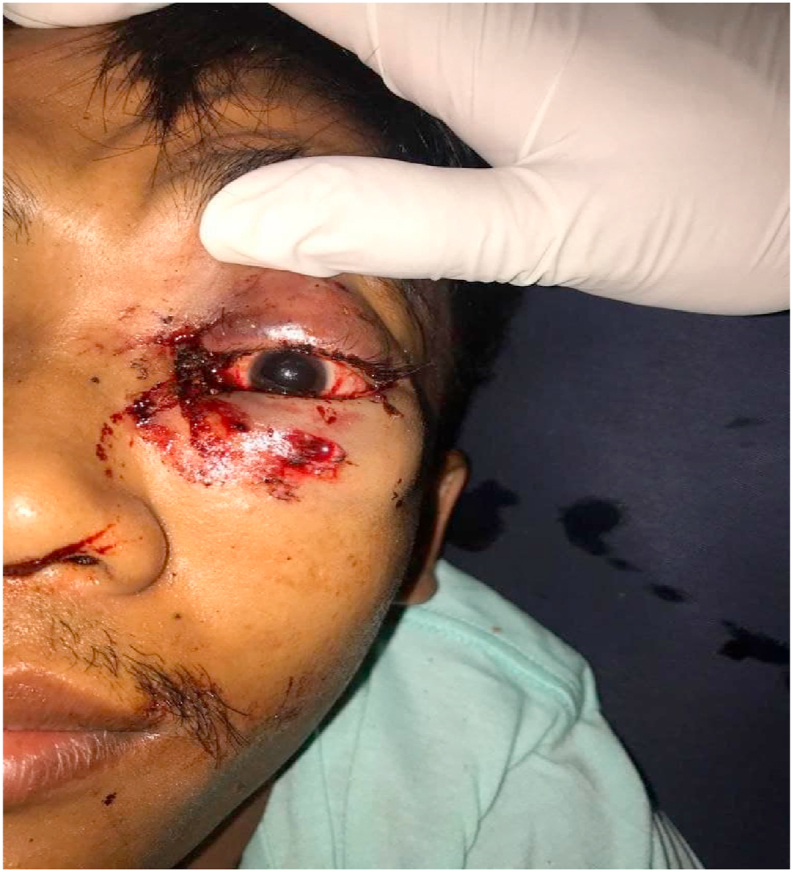
Bleeding and minor laceration medial canthus with hematoma in the left upper eyelid.

A 27-year-old male came to the emergency department with blood came from his left eye after falling from a motor vehicle and struck the stack of tree branches. The patient was fully conscious and had only a minor hematoma and a small wound in the left medial cantal region. Neurological examination revealed no decrease in mental status (GCS 15) with visual acuity markedly decreased. The patient could only differentiate light perception with different sizes of the right and left pupil. Ocular motility was markedly decreased (Fig. 1).

**Fig. 2:**
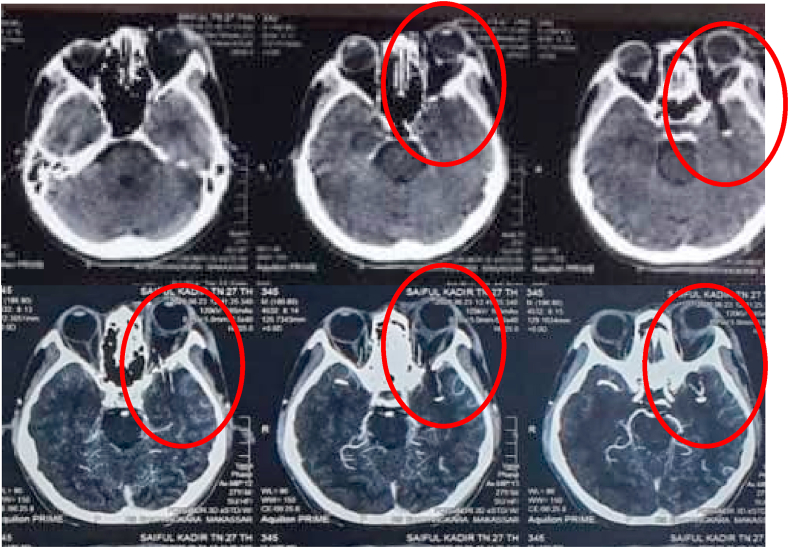
Noncontrast and contrast head CT scan showed a linear-shaped hypodense foreign object in left medial orbit penetrate to the left temporal fossa, no contusion and cerebral edema.

No sign of meningeal irritation was observed, the function of trigeminal and facial nerves was preserved. Unfortunately, further ophthalmologic examinations were not done. A non-contrast head CT was performed and reveal a linear-shaped foreign body, air mimicking that penetrated the medial orbit plane to the left temporal fossa. From the brain window aspect, there was no cerebral edema and fracture seen (Fig. 2).

**Fig. 3:**
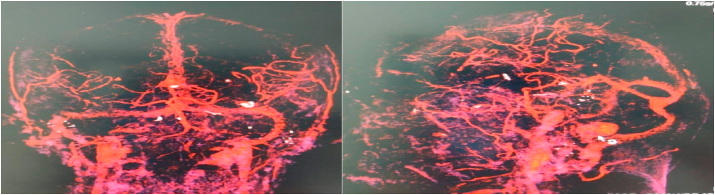
CT Angiography showed no vascular injury or vascular near to the foreign body.

A CT angiography was also performed to see if there was a vascular injury and vascular near the foreign body or traumatic vascular aneurysm(Fig. 3).

**Fig. 4:**
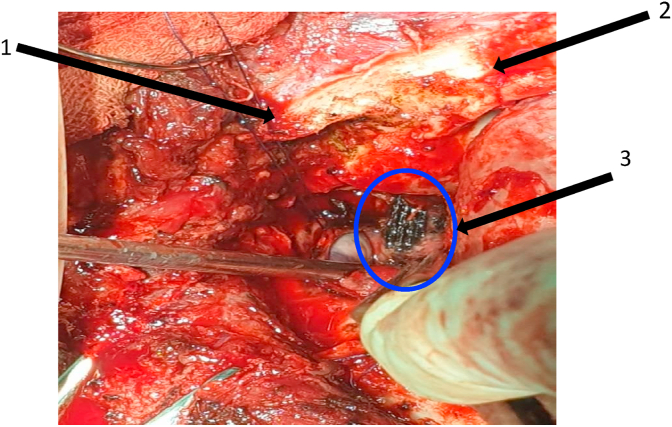
Wooden foreign body was seen in the left temporal fossa. (1) frontozygomatic arch, (2) Frontal, (3) wooden foreign body.

**Fig. 5:**
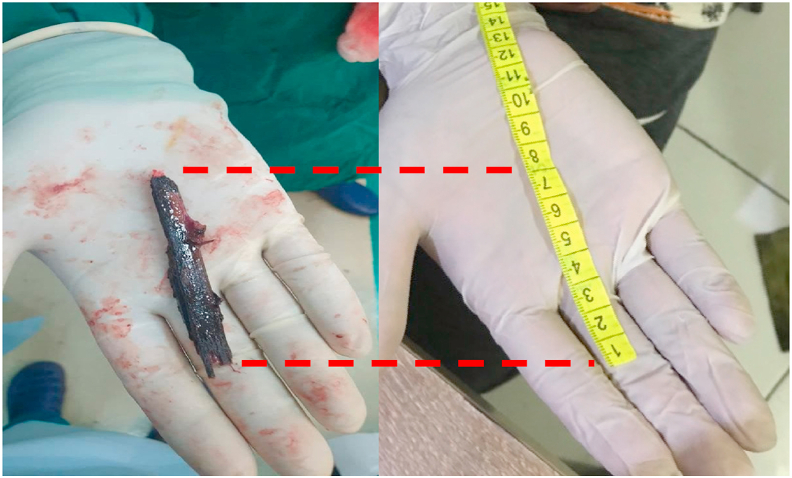
Wooden foreign body was seen after retracted from intracranial with length approximately 7 cm.

The patient underwent a craniotomy on the temporobasal approach. Neurosurgeon et al. decided to use the temporobasal approach not only because it was the closest approach to foreign body position but also to get a clear visualization of the foreign body through the intracranial cavity and also it is good for cleaning up the wood fragments that entered the intracranial cavity. The patient was lying in the supine position and temporofrontal muscles were incised layer by layer. Craniotomy was performed at the temporal basal region and the dural was incised. The brain parenchymal was retracted and the foreign body was seen (Fig. 4, Fig. 5).

**Fig. 6:**
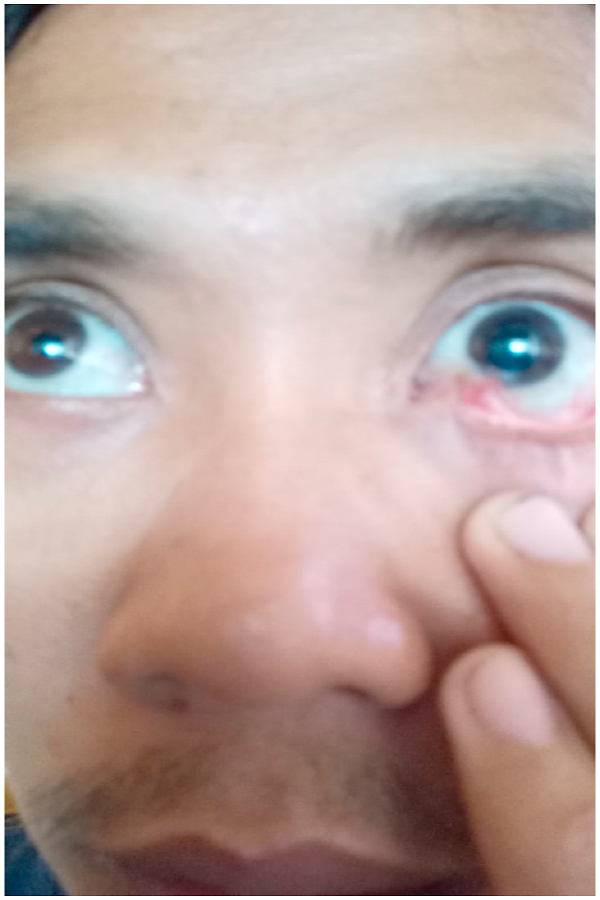
A membrane growth from medioinferior site as it partially covers the lower part of the left eye (mirroring effect due to a selfie taken mode photo), the pupil size between the right and left eye are unequal.

Small vessels were coagulated and the foreign body was retracted from the outside slowly. After that, exploration and irrigation of the wood particles with bleeding control were done meticulously. Postoperatively, the patient was admitted to ICU where broad-spectrum antibiotic was administered. No signs of infection were detected and the patient was fully recovered and discharged after 5 days. However, the patient only came to visit the ophthalmologist for late orbital complications which seen in the picture and yet never came to the neurosurgery department for further follow-up so it was difficult to detect any late intracranial complication. However, we still could contact the patient and asked several questions related to his condition. The patient said that he could not move his left eye to the lateral side, and it seemed the size of both pupils were unequal (Fig. 6).

## 3 Discussion

TOPI was relatively rare. It can be caused by high-speed projectile foreign bodies to low-energy trauma (which is rarer), and account for 24% of penetrating head injuries in adults and approximately 45% in children and who are prone to trauma while playing games improperly [3,5,6]. Traumatic brain injury has higher mortality and morbidity than blunt trauma due to Diffuse Axonal Injury is prevalent in traumatic brain injury also it can cause damage to the orbit (and structures surrounding it), vascular, and brain that can lead to the blindness, neurological deficits even death [[1], [7]]. The structural characteristics of the orbit play an important role in the pathogenesis of orbital injuries with intracranial penetration. The orbit is in a shape of a horizontal pyramid on a posteromedial axis. This shape tends to deflect objects entering the orbit toward the apex where the superior orbital fissure and optic foramen may provide a passage to the intracranial contents [8]. There are three usual routes through which the foreign body (FB) can penetrate intracranially; through the orbital roof route, superior orbital fissure, and the optic canal. The first one is the most common cause due to the fragility of the superior orbital plate of the frontal bone in the anterior cranial fossa floor hence make it vulnerable to cause intracranial injury in such trauma as seen in this case. This commonly occurs when one falls onto objects, that are directed in an upward direction, fracturing the thin frontal bone and causing a frontal lobe injury. The second most common site is through the superior orbital fissure (SOF) whereby foreign bodies occasionally reach the brainstem through the cavernous sinus, thereby resulting in a serious injury. According to Turbin criteria, this case is zone 3b. Interestingly, the trajectory and the site of CNS injury didn't follow the Turbin common pattern in zone 3b [9]. In this case markedly decreased visual acuity and extraocular movement in the left eye, unequal size of both right and left pupils and in the left eye without altering mental status mostly because of the injury of the optic nerve and oculomotor nerve (including muscles compartment) without reaching the brainstem. The severity of the damage, however, depends on the size and shape of the stab tool, on its trajectory, force, and entry point^3 5^. Patients may present in an unconscious state, and sometimes, a patient who is conscious at the time of presentation can deteriorate very fast. This indicates that the GCS score at admission is not always a good predictor. However, our patient was fortunate to be conscious throughout the hospital course. Clinically, a major neurological symptom may be absent on the first examination in children in particular when the penetration is not via the superior orbital fissure or optic canal and the eyeball is not injured [10].

In clinical practice, physical examination, including full neurological and/or ophthalmological examinations is important in the diagnosis and appropriate treatment of any patient diagnosed with transorbital intracranial trauma because injuries involving a wooden foreign body are even scarcer than those involving other materials such as metal or glass and may pose unusual diagnostic and therapeutic challenges. Due to dry wood's air-filled porous microstructure, it mimics pneumocephalus on CT scan (both in soft and bone windows) therefore raising a radiological diagnostic challenge in intracranial wood penetrating injury. Helpful differentiating feature of wooden foreign body from pneumocephalus is the typical geometric shape of the foreign body and different attenuation values-dry wood measures typically in the −100 to −170 HU range while pneumocephalus measures in the −600 to −1000 range. And yet, CT attenuation values vary with time as water content of the wood can change with time and so a freshly cut wood which has a relatively high water content may mimic soft tissues in CT images. Also when the setting were altered by increasing the window width, the foreign body were more easily identified [11,12]. Likewise in this case, CT scan was performed only several hours after trauma but combining with corresponding history of traumatization make no doubt about the impalement injury as it remains as primary choice in emergency department [3,5,13]. MRI is a safe modality (as long as no metallic foreign body was suspected) with higher sensitivity compared to CT due to it’s ability to detect wood foreign body and soft tissue.^3 5^ CT angiography or MR angiography, is indicated when there is evidence of a possible vascular injury, either by the location and trajectory of the foreign body or evidence of a hematoma on CT scanning If there is suspicion of a vascular injury, angiography should also be performed to evaluate for the presence of a traumatic aneurysm, which can develop soon after a penetrating injury. Other indications for an early angiographic evaluation are fracture of the greater wing of the sphenoid and examination findings consistent with CN injury, both of which suggest possible injury to the middle cranial fossa or the cavernous sinus. Therefore, combination of multiple modality of imaging e.g CT, MRI, and CTA or DSA are essential for correct diagnosis and surgical planning^5 14^

Management should includes multidiciplinary approach (at least by ophtalmologist and neurosurgeon) for satisfactory result [15,16]. Altough there is still no standardized treatment for these events because different injury pattern for each scenario. However, certain principles can be employed to almost any case in order to optimize patient outcome. Early removal of the entire foreign body, removal of bone fragments, focal debridement, decompression of neurovascular structures, hemostasis, and dural repair are key goals of surgery [17]. The removal of the wooden material and intracranial hematoma (if any) is essential. If not removed in time, serious complications like discharging sinus and fulminant meningitis may occur days, months, or even years after initial trauma. Miller et al. reported that infection was a complication in 64% of their 42 cases of intracranial wooden foreign bodies, in spite of the use of antibiotic agents. Brain abscess occurred in 48%, and the total mortality rate was 25%. So the early removal of foreign body with thorough irrigation is essential [8,10] fortunately in our case there was no clinical sign of infection occur postoperatively which in this case, the operation of removal wood foreign body was held about 48 hours after onset.

The general principle guiding surgical treatment of such patients is preferred to be a direct visualization of the object and extent of the injury also called “open and see”. Classically, three surgical approaches have been described to remove a foreign body: frontotemporal craniotomy, subcranial craniotomy, and anterior orbitotomy. A transorbital or transcranial approach can be chosen depending on the location of the fragment [18]. Frontotemporal and subcranial craniotomies can be done with a bicoronal incision and flap. Craniotomy is indicated only when intracranial injury is anticipated as decompression of neural structures and repair of bony and dural defects is possible. We used a frontotemporal approach in this patient as it is used when a wide exposure is necessary due to extensive injury and/or involves the middle fossa. The subcranial approach is used for anterior fossa injuries, which require wide exposure. Anterior orbitotomy is used to repair orbital roof fractures if there is no evidence of vascular injury on CT scans. Compared to craniotomy, orbitotomy is faster, less invasive, and has a shorter recovery time. A fourth approach is also documented: the transpalpebral approach to repair injuries sustained from traumatic orbital roof fracture that result in dural lacerations in the area of the orbital roof. Their advantages are that it is minimally invasive, shorter hospital stay, and with good cosmetic results [10]. some authors also reported an occipital craniotomy with good result [1]. Ying Yao et al. also reported a percutaneus endoscopic technique via minimally invasive approach that showed excellent outcome [14].

Immediate complications include intracerebral hematoma, cerebral contusion, intraventricular hemorrhage, pneumocephalus, brain stem injury, and cerebrovascular injuries. According to some study, infection can appear as early or late complication, but it seem happen sooner in wooden foreign body than in metallic object so early radical debridement and removal of the retained fragment are mandatory to prevent potentially fatal infectious complications. Chibbaro et al. even reported one case of TOPI with wooden foreign body causing intracerebral abscess in spite of removal of the foreign body was already done [[18], [19], [20]], so that broad spectrum antibiotics should always be administered in every TOPI patients. Some authors even reported the administration of broad-spectrum intravenous antibiotic until few weeks and can be changed according to the result of microbiology test. In our case the patient only given antibiotic until 6 days and yet showed no clinical sign of infection. Massive irrigation and meticulous debdridement also took a part. We didn't give anticonvulsant therapy in this patient as a prophylactic altough risk of postoperative seizure is as high as 30–50%, of which 10% appear within first week of trauma because most common cause of seizure are compound depressed fracture and intracranial infection which not seen in this patient. Early surgical intervention might decrease the chance of formation of scar tissue and hence of an epileptic focus [21]. However a further investigation and follow-up must be taken due to some late complications can occur like traumatic aneurysm, the possibility of seizure in late onset as reported in Chunhua et al. study that reported recurrent seizures after 30 years of pencil retained in temporal lobe at the setting of TOPI [20,22]. The writing of this script follows the rules of the SCARE 2020 Guideline. The work has been reported in line with the SCARE 2020 [23].

## 4 Conclusion

Transorbital Intracranial Penetrating Injury (TOPI) is a rare case in civillian practice, moreover the one that caused by wooden foreign body. Early removal of wooden foreign body that penetrate to the intracranial by looking directly at the intracranial cavity is mandatory to prevent bleeding and cleaning wood chips. It should be involved multidisciplinary approach in order to get optimal result and avoid the fatal complication both neurologically or ophtalmologically.

## Provenance and peer review

Not commissioned, externally peer-reviewed.

Written informed consent was obtained from the patient for publication of this case report and accompanying images. A copy of the written consent is available for review by the Editor-in-Chief of this journal on request.

## Funding

None.

## Ethical approval

Obtained.

## Sources of funding

No funding or sponsorship.

## Author contribution

WYD, AHZ, AAI, RHA, PRI wrote the abstract, introduction, case, discussion, conclusion. WYD, AAI, RHA, PRI performed critical edits and final revision, figures.

## Consent

Obtained

## Registration of Research Studies

1.Name of the registry: NA

2.Unique Identifying number or registration ID: NA

3.Hyperlink to your specific registration (must be publicly accessible and will be checked): NA

## Guarantor

Rohadi Muhammad Rosyidi.

## Declaration of competing interest

None.
